# Tracking costs of virulence in natural populations of the wheat pathogen, *Puccinia striiformis *f.sp.*tritici*

**DOI:** 10.1186/1471-2148-9-26

**Published:** 2009-01-30

**Authors:** Bochra Bahri, Oliver Kaltz, Marc Leconte, Claude de Vallavieille-Pope, Jérôme Enjalbert

**Affiliations:** 1 UMR BIOGER CPP, INRA Agro-Paris-Tech, BP01, 78850 Thiverval-Grignon, France; 2 UPMC UnivParis 06, Laboratoire de Parasitologie Evolutive – UMR 7103, 7 quai St-Bernard, 75252 Paris, France; 3 Institut des Sciences de l'Evolution – UMR 5554, Université Montpellier 2, Place E. Bataillon (CC065), 34095 Montpellier Cedex 05, France

## Abstract

**Background:**

Costs of adaptation play an important role in host-parasite coevolution. For parasites, evolving the ability to circumvent host resistance may trade off with subsequent growth or transmission. Such costs of virulence (*sensu *plant pathology) limit the spread of all-infectious genotypes and thus facilitate the maintenance of genetic polymorphism in both host and parasite. We investigated costs of three virulence factors in *Puccinia striiformis *f.sp.*tritici*, a fungal pathogen of wheat (*Triticum aestivum*).

**Results:**

In pairwise competition experiments, we compared the fitness of near-isogenic genotypes that differed by a single virulence factor. Two virulence factors (*vir4*, *vir6*) imposed substantial fitness costs in the absence of the corresponding resistance genes. In contrast, the *vir9 *virulence factor conferred a strong competitive advantage to several isolates, and this for different host cultivars and growing seasons. In part, the experimentally derived fitness costs and benefits are consistent with frequency changes of these virulence factors in the French pathogen population.

**Conclusion:**

Our results illustrate the variation in the evolutionary trajectories of virulence mutations and the potential role of compensatory mutations. Anticipation of such variable evolutionary outcomes represents a major challenge for plant breeding strategies. More generally, we believe that agro-patho-systems can provide valuable insight in (co)evolutionary processes in host-parasite systems.

## Background

Central to many concepts in evolutionary biology is the idea that adaptation is not cost-free [1,2]. Costs arise if adaptation in one trait is opposed by a negative correlated response to another trait. Such trade-offs between fitness components can influence life-history evolution, ecological specialisation and more generally, the maintenance of genetic diversity [3].

Costs of adaptation also play an important role in host-parasite coevolution. In the host, costs of resistance refer to trade-offs between resistance and other fitness-relevant traits, conferring a selective disadvantage to resistant genotypes in the absence of the parasite. The corresponding costs in the parasite, referred to as 'costs of virulence' in the plant-pathogen literature, are trade-offs between the capacity to establish an infection and other parasite characters (e.g., within-host growth, production of transmission stages [4]). These costs are considered major ingredients of the coevolutionary process because they can prevent the spread to fixation of all-resistant host or all-infectious parasite genotypes and thereby preserve genetic diversity in both host and parasite populations [5-8].

While costs of resistance have been investigated in various host-parasite systems (plant: [9,10]; (cyano)bacteria: [11,12]; protozoa: [13]; drosophila: [14]), still little is known about costs of virulence and their role in shaping genetic structure and coevolutionary dynamics. Historically, this issue has first been addressed in plant-pathogen interactions [15,16]. Here, virulence refers to the ability of the pathogen to circumvent host resistance and establish an infection. This ability often arises from mutational loss of function of genes that would otherwise elicit a defense reaction of the host [17]. However, this advantage of malfunctioning can turn into a disadvantage if these genes are essential for subsequent development of infection. With the advent of molecular techniques, investigation of the precise genetic and physiological basis of costs of virulence has become possible. Induced mutagenesis of genes implicated in plant-pathogen recognition revealed that at least some of these genes are involved in within-host replication, pathogenicity or spore production [18-20]. Thus, loss of function of these genes will allow infection, but also impose a straightforward cost for subsequent development.

Given their intrinsic fitness costs, selection should eliminate unnecessary virulence genes from the pathogen population and favour pathotypes with particular virulence genes that match the resistance structure of the host population [21]. Thus, in classical models of plant-pathogen coevolution, changes in the frequencies of resistance and virulence genes are essentially determined by the fitness trade-offs associated with these genes [6,15]. While more recent theoretical developments balance the importance of cost with other biological features [22,23], empirical and experimental evidence of these basic theoretical concepts is still scant. Natural populations of various pathogens often harbour genotypes carrying different numbers of virulence alleles. For pathogens of crop plants, withdrawal of a particular cultivar sometimes leads to the subsequent decrease in frequency of the genotypes carrying the corresponding virulence allele [24,25], consistent with the idea of selection against unnecessary and costly virulence alleles. In some cases, molecular analysis suggests that costs of virulence contribute to the maintenance of genetic polymorphism in the pathogen [26]; in yet other cases, elimination of the function of virulence genes seems to be cost-free, suggesting that fitness costs are not universal and depend on the gene or the pathogen considered.

Only few experimental studies compared the relative fitness of naturally occurring pathogen genotypes that carry different numbers of virulence alleles. Thrall and Burdon [27] detected a negative correlation between virulence and spore production for the plant pathogen *Melampsora lini*. This trade-off may explain why relatively avirulent strains dominated susceptible host populations. Furthermore, for different plant-pathogen systems, competition experiments compared pathogen genotypes with different numbers of virulence alleles. Some of these studies demonstrated a selective advantage of simpler over complex genotypes, at least in the greenhouse [[19,27,28], but see [29]]. Typically, however, these experiments did not control for the genetic background of the competing strains and it is possible that fitness differences were caused by other genes [30].

Here, we investigated costs of virulence in the yellow rust fungus, *Puccinia striiformis *f.sp.*tritici *(*PST*), one of the most damaging wheat pathogens worldwide [31]. This clonally reproducing pathogen rapidly evolves through stepwise mutational acquisition of new virulences [32,33], and the resulting arms race between plant breeders and the pathogen is characterised by frequent and rapid resistance breakdowns [34]. In North-Western Europe, the fitness advantage of being able to grow on a resistant cultivar is so strong that a new virulent mutant pathotype can replace an existing dominant pathotype within 2–3 years [33,35]. Costs of virulence potentially slow down resistance breakdowns or at least limit the severity of epidemics. Therefore knowledge of these costs can help plant breeders to better understand evolutionary change in the pathogen and to develop more efficient strategies of resistance management.

We performed competition experiments using pathogen genotypes that differed by a single virulence gene not needed for infection. This is analogous to measuring costs of resistance in the absence of the parasite [4,10]. If the virulence gene is costly, its carriers should be at a competitive disadvantage. We tested this hypothesis for different naturally occurring virulence genes. The specific novelty of this study is that we explicitly accounted for genetic background by competing genotypes with identical (or nearly so) profiles of molecular markers (AFLP). Because this pathogen is clonal, identical profiles indicate very close phylogenetic relationships. Further, competition assays were carried out both in the climatic chamber and in the field, on different wheat cultivars and in different years. We confront our results with changes in the frequencies of these virulence factors in natural populations over the past 20 years [34], and with changes in aggressiveness in a collection of isolates from this period.

## Results

### 1. Competition experiments

The 16 isolates used in these experiments were collected from naturally infected fields in France between 1989 and 1997. Competition experiments were carried out between pairs of fungal isolates with identical AFLP marker profiles (see methods). These isolates also had identical virulence gene profiles, except that one of the two isolates carried one additional virulence gene (Table 1). The isolate with the additional virulence gene will be referred to as *'virulent' *(*vir*), the one without it as *'avirulent' *(*Avir*). By definition, virulent isolates are fully infective (fitness = 1) on resistant plants, whereas avirulent isolates cannot infect these plants (fitness = 0). Remember that '*virulent' *here is used in the plant pathology sense, i.e., it refers to the capacity to infect a host; to describe the damage done to the host, we use the term 'aggressiveness' [36]. In the present experiments, host varieties were chosen such that both types of isolates were infectious, that is, the additional virulence gene was redundant with respect to infectivity of the isolate and thus potentially costly. We tested the effects of three virulence genes *vir9, vir6 *and *vir4 *in field experiments, with an additional focus on *vir9 *in controlled conditions.

**Table 1:** Identity of pathogen isolates in the pairwise competition experiments

**Virulence**	**Pathotype^1^**	**Virulence factor profile^2^**								**Isolate**	**Reference^3^**	
*Avir9*	109E141-*Avir17*	*1*	*2*	*3*	*4*	*6*	-	*SD*	-	*i1*	J89138	A					F
										*i2*	J89110		B			E	
										*i3*	J8861M			C			
										*i4*	J89122				D		
*vir9*	237E141-*Avir17*	*1*	*2*	*3*	*4*	*6*	***9***	*SD*	-	*i5*	J89121	A				E	
										*i6*	J89137		B				F
										*i7*	J9521M1			C			
										*i8*	J9522M2				D		

*Avir4*	173E140-*vir17*	*1*	*2*	*3*	-	*6*	*9*	*SD*	*17*	*i9*	J99198	G					
										*i10*	J0085F		H				
*vir4*	237E141-*vir17*	*1*	*2*	*3*	***4***	*6*	*9*	*SD*	*17*	*i11*	J01144B-M1	G					
										*i12*	J02022		H				

*Avir6*	169E136-*vir17*	*1*	*2*	*3*	-	-	*9*	*SD*	*17*	*i13*	J9791M	I					
										*i14*	J9782		J				
*vir6*	173E140-*vir17*	*1*	*2*	*3*	-	***6***	*9*	*SD*	*17*	*i15*	J02022	I					
										*i16*	J02055C		J				

Three virulence factors (*vir9, vir4, vir6*) were tested, with 16 natural isolates *(i1-i16) *of *P. striiformis *f.sp. *tritici*. For each of the 10 pairs (*A-J*), the two competing isolates had (nearly) identical AFLP profiles and identical virulence factor profiles, except for the virulence factor to be tested (marked in bold). The isolate carrying the additional virulence gene is referred to as *virulent (vir)*, the one without it as *avirulent (Avir)*. ^1 ^Nomenclature determined by reactions on a set of European and world differential cultivars; ^2 ^*SD *refers to virulence on the cultivar *Strubes Dickkopf*; ^3 ^Lab references of each isolate. The two numbers after the "J" denote year of sampling of the isolate.

#### vir9 pathotype

**Figure 1:**
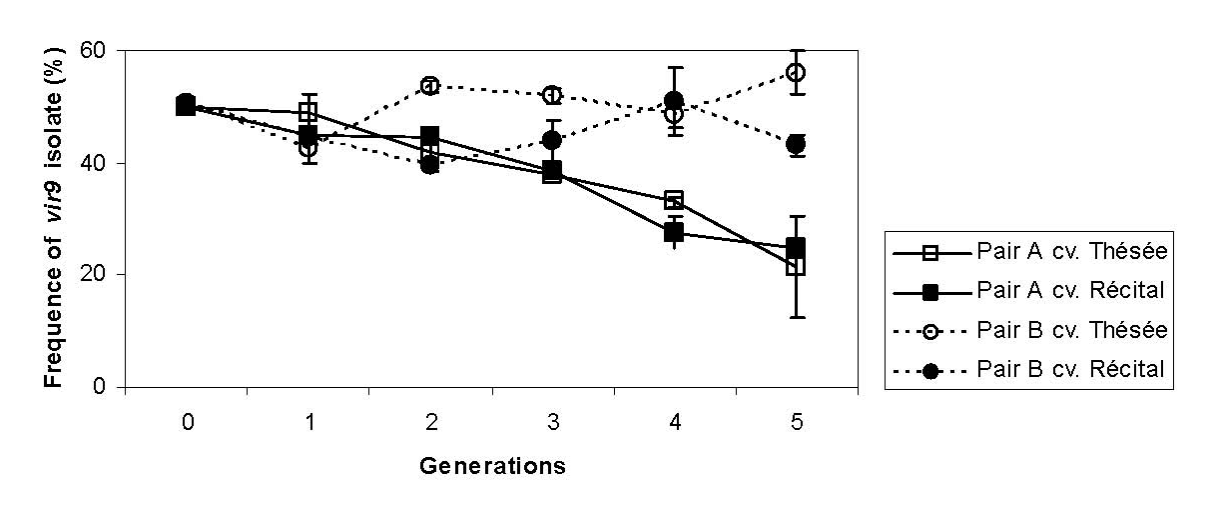
**Competitive success of *vir9 *isolates in the climate chamber experiment**. Mean (± S.E.) frequencies of *vir9 *isolates relative to *avir9 *isolates over the course of 5 pathogen generations in pairwise competition experiments. Two A *vir/vir *pairs (*A*, *B*) were competed on two host cultivars (*Thésée*, *Récital*). Each point represents the mean and S.E. calculated over 2 independent replicates.

In a climate chamber assay, we competed two independent pairs (*A, B*, see Table 1) of a *virulent *and an *avirulent *isolate, initially in a 50:50 mix. For pair *A*, the relative frequency of the isolate (*i5*) with the additional virulence allele decreased by 50% over the course of five infection cycles, and this on both host cultivars (Figure 1). For pair *B*, deviations from the 50:50 ratio were less pronounced and differed between host cultivars (pair × host cultivar × time interaction: F_4,16 _= 3.67, p = 0.0263; repeated measure ANOVA).

**Figure 2:**
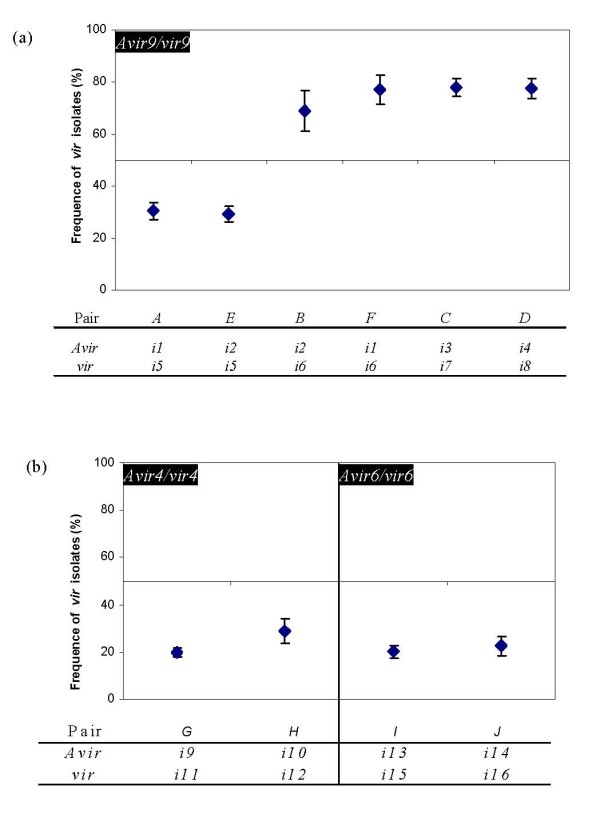
**Competitive success of virulent isolates in field experiments**. Mean (± S.E.) frequencies of *vir9 *(a), *vir4 *and *vir6 *(b) isolates relative to their corresponding *avirulent *(*Avir*) isolates, as determined in pairwise competition experiments. Competing pairs (*A-J*) of *virulent *(*vir*) and *avirulent (Avir) *isolates (*i1–i16*) were started at an initial 50:50 ratio and the relative frequency of the *vir *isolate measured at the end of the growing season. Each point represents the means and S.E. calculated over 2 years and 2 host cultivars. For all 10 pairs, final frequencies were significantly different from the initial 50:50 ratio (t_7 _> 3.49; p < 0.0101).

In field competition assays, the relative frequencies of *avirulent *and *virulent *isolates at the end of the growing season varied strongly among four independent pairs (*A, B, C, D*; Figure 2a, Table 2). Starting from an initial 50:50 ratio, the relative frequency of the *virulent *isolate (*i5*) in pair *A *decreased significantly by about 50%, just like in the greenhouse assay. In contrast, virulent isolates (*i6, i7, i8*) doubled in frequency in the three other pairs (*B*, *C*, *D*). With one exception (pair *B*, 2005, on the *Récital *cultivar), these patterns were consistent over growing seasons (2005, 2006) and host plant cultivars (*Récital*, *Thésée*) (Table 3). Thus, in one case (*i5*), the isolate with the additional virulence was less competitive than its isogenic counterpart; in the three other cases, it was more competitive (*i6, i7, i8*). The selective advantage or disadvantage of these *virulent *isolates was in the order of 15–20%.

**Table 2:** Success of virulent isolates in pairwise competition experiments in the field

***Avir/vir *competition**	**Pair**	***Avir***	***vir***	**Year 1**		**Year 2**		**Selective value of *Avir***
					
				Cultivar 1*	Cultivar 2**	Cultivar 1*	Cultivar 2**	
*Avir9/vir9*	*A*	*i1*	*i5*	38 ± 2.7	26 ± 0.2	24 ± 1	32 ± 10.2	0.15
	*E*	*i2*	*i5*	-	-	26 ± 9.8	32 ± 0.2	0.16
	*B*	*i2*	*i6*	80 ± 3.5	46 ± 6.4	74 ± 0.2	76 ± 0.3	-0.17
	*F*	*i1*	*i6*	-	-	71 ± 0.9	83 ± 0.3	-0.27
	*C*	*i3*	*i7*	85 ± 4.4	70 ± 11.6	74 ± 5.3	83 ± 3.3	-0.29
	*D*	*i4*	*i8*	83 ± 8.6	72 ± 5	70 ± 3.2	85 ± 5.1	-0.28

*Avir4/vir4*	*G*	*i9*	*i11*	-	-	22 ± 0.1	18 ± 0.9	0.24
	*H*	*i10*	*i12*	23 ± 0.8	44 ± 6.3	28 ± 5.1	20 ± 0.3	0.17

*Avir6/vir6*	*I*	*i13*	*i15*	17 ± 1.4	14 ± 1.9	25 ± 6.1	25 ± 0.9	0.24
	*J*	*i14*	*i16*	29 ± 14.5	28 ± 9.9	20 ± 2.2	12 ± 1.9	0.22

Mean (± S.E.) frequencies (%) of *virulent *(*vir) *isolates relative to *avirulent *(*Avir*) isolates, measured at the end of the growing season. Different pairs (*A-J*) were set up for each of 3 virulence genes (*9, 4, 6*). Each pair was started from an initial 50:50 ratio and competed on two host cultivars and in two field seasons (year). Selective values of *Avir *isolates were estimated according to Leonard [28] and represent averages over cultivars and years. *Cultivar 1 correspond to cv. *Thésée *for *Avir9/vir9 *and cv. Audace for *Avir4/vir4 *and *Avir6/vir6*, respectively. ** Cultivar 2 correspond to cv. *Récital *for *Avir9/vir9 *and cv. Baltimore for *Avir4/vir4 *and *Avir6/vir6*, respectively.

**Table 3:** Statistical analysis of competitive success of *vir9 *isolates in the field competition experiments

**Source**	**denm**.	**DF**	**MD**	**F**	**Pr > F**
Year	1+3–5	1,1	1.13	0.09	0.8072
Cultivar	2+3–5	1,1	10.71	0.88	0.5203
Pair	1+2–5	3,4	47.80	15.57	0.0114
Year*Pair (1)	4	3,3	1.84	3.23	0.1807
Cultivar*Pair (2)	4	3,3	1.45	2.54	0.2321
Year*Cultivar (3)	4	1,3	11.36	19.93	0.0209
Year*Cultivar*Pair (4)	5	3,16	0.57	0.57	0.6438
Residual (5)		16	1.00	-	-

Based on a logistic regression, Analysis of Deviance tested for effects of pair (*Avir/vir*), host cultivar and year on final frequencies of *vir9 *isolates. Analogous to Analysis of Variance, mean deviances (MD = 2.log-likelihood ratio/DF) were used to calculate quasi-*F *values. The denominator (denm.) column indicates the error terms used for the *F*-tests; where necessary, linear combinations of different model factors were made [69]. The DF column indicates the numerator and denominator degrees of freedom. All factors were considered as random effects. Model fitting was analogous to SAS-type II model fitting [68].

With two additional competition pairs (*E*, *F*) we were able to compare all possible A *vir*/*vir *combinations between the *i1, i2, i5 and i6 *isolates. Analysis of this set of combinations (pairs *A*, *B*, *E*, *F*) revealed a significant effect of *virulence *identity (*i5 *vs. *i6*; F_1,8 _= 142.61, p = < 0.0001), but no significant interaction between *avirulence *and *virulence *identity (F_1,8 _= 0.05, p = 0.832). That is, the *i5 *isolate was less competitive and the *i6 *more competitive, regardless of the identity of the *avirulent *counterpart in the mix.

#### vir4 and vir6 pathotypes

For the *vir4 *and *vir6 *pathotypes, four pairs of *avirulent *and *virulent *isolates were tested (Pairs *G*-*J*, Table 2) under field conditions. For all *virulent *isolates, we observed a significant decrease in the frequency (*vir4*: t_7 _= 12.76, p < 0.0001; *vir6*: t_5 _= 5.19, p = 0.0035) (Figure 2b, Table 2). Typically, their relative frequencies dropped from initially 50% to around 20–30% within a growing season, although this effect varied to some extent between years (*vir4*: year × cultivar interaction: F_1,6 _= 17.4, p = 0.0059) and host cultivars (*vir6*: year × pair interaction: F_1,8 _= 7.44, p = 0.0260). Thus, in all four pairs tested, the isolate with the additional virulence was substantially less competitive, with a selective disadvantage of 17–24%.

### 2. Sporulation test and genetic analysis of the vir9 pathotype

**Figure 3:**
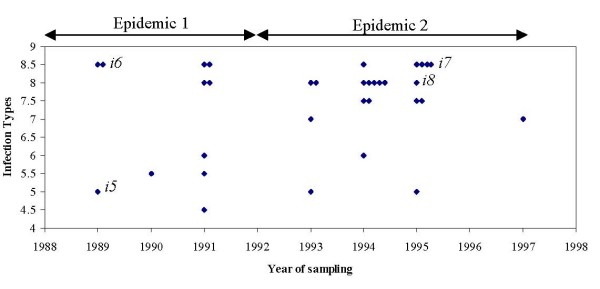
**Time series of aggressiveness of *vir9 *isolates**. Infection types on *Clement *seedlings of 33 isolates carrying the *vir9 *gene and collected from French populations during two epidemic periods between 1989 and 1997. Large values of infection type indicate high levels of sporulation and thus high aggressiveness. Each point represents the mean of two independent measurements. Four isolates (*i5–i8*) were used in the field competition experiments.

We further investigated temporal changes in the genetic composition of the French population for the *vir9 *pathotype. To this end, we characterised AFLP profiles and measured aggressiveness of 33 *vir9 *isolates collected between 1989 and 1997. Despite thorough screening of 39 primer combinations, no variation in the neutral markers was detected, indicating that these isolates all belonged to the same clonal line of descent. In contrast, isolates differed significantly in their aggressiveness, as measured by the infection type (IT) index (F_32,71 _= 8.39, p = < 0.0001; Figure 3). This visual classification index was highly correlated with quantitative measurements of sporulation rate (r = 0.75, n = 185, p = < 0.0001), showing an up to 5-fold difference in sporulation rate between isolates. After the resistance breakdown of the *Yr9 *cultivar in 1989, a first epidemic occurred between 1989 and 1991, a second between 1993 and 1997. In the collection, the variance in IT significantly decreased between the first and the second epidemic period (F_1,31 _= 5.1880, p = 0.0298 in O'Brien), with a concomitant 9% increase in the mean IT index. We found a considerably higher frequency of heavily sporulating isolates (IT index > 7) during the second epidemic (χ^2 ^(1) = 4.04, p = 0.044; Figure 3). These changes suggest a response to natural selection in aggressiveness during the two epidemics. Finally, note the link between performance in the competition assays and sporulation rate: the less competitive *i5 *isolate had a much lower sporulation rate (0.17 ± 0.08 mg spores/chlorose) than the three more competitive isolates (*i6, i7, i8*: 0.37–0.53; see also Figure 3).

## Discussion

### Evidence for costs of virulence

For five of the 8 *virulent *isolates tested, our experiments demonstrated a substantial cost of carrying an additional virulence gene, and this for different genes and different genetic backgrounds of the same gene. Despite some variation between years and cultivars, these costs appear to be quite robust and repeatable. In particular, the additional tests (pairs *E*, *F*) showed that the low performance of the *virulent *isolates was independent of the identity of the *avirulent *competitor.

Genetic variation in fitness detected in the laboratory does not always reflect the degree of variation expressed under field conditions. Under natural conditions, stress can increase the genetic variance in fitness [37], while confounding environmental effects might limit its detection [38]. Here, the field assays revealed even stronger fitness differences between the competing isolates (Figure 2). The selection coefficients (13%–22%) are similar to those from laboratory studies on other organisms [39-41] and indicate potentially strong selection against these virulence genes, if redundant. The most likely cause of this competitive disadvantage is a reduction in the ability to develop on the host and produce spores. This type of cost has been detected in other pathogens [39-43] and at least for the *vir9 *virulence gene, we have direct evidence that costs are due to low sporulation.

### Compatibility with epidemiological history of natural PST populations

In agricultural host-parasite arms races, farmers typically plant one or a few host cultivars over large geographic areas. This imposes strong, uniform selection on the pathogen to evolve the ability to attack these host genotypes. In the case of *PST*, the necessary virulence genes to overcome resistance can rapidly arise through mutation in large clonal populations. Once a resistance breakdown has occurred, cultivars are withdrawn and replaced by new resistant cultivars, developed by plant breeders. Indeed, one of the to date most convincing examples of such a semi-natural coevolutionary arms race has been demonstrated for the PST-wheat system [34], and it involves the virulence genes (*vir4, vir6, vir9*) studied in our experiments. With the replacement of susceptible cultivars, the *vir6 *and *vir9 *genes have become redundant at some point during the past 15 years (*vir4 *has always been redundant). Thus, if these genes impose fitness costs, they should have decreased in frequency in natural pathogen populations[21].

For the *vir6 *virulence gene, this seems to be the case. The competition experiment revealed a strong selective disadvantage of the virulent *vir6 *isolates, and this is consistent with three declines in the frequency of the *vir6 *gene, when cultivars carrying the corresponding resistance gene (*Yr6*) were retracted [34]. In contrast, the fitness costs of the *vir4 *virulence gene detected in the competition experiment does not match with its epidemiological history. The *vir4 *gene appeared in Europe already more than 20 years ago[34,44] and all contemporary pathotypes carry it. Interestingly, the majority of plant varieties used in France in the past 30 years did not carry the corresponding *Yr4 *resistance gene and thus the *vir4 *gene must have been mostly redundant. Given the clonal evolution of *PST*, it is possible that this ancestral *vir4 *gene has spread to fixation by hitch-hiking with new, selectively advantageous virulence genes. Originally, the *vir4 *gene may have immigrated from the UK, where the *Yr4 *resistance gene was present in several cultivars in the past (e.g., Maris Beacon, Rapier, Avalon or Brimstone [45-47]). In fact, the *Yr4 *has also been postulated for certain Danish wheat cultivars [48]. This suggests that the *vir4 *gene may even have a selective advantage at a larger, European scale and that simple hitch-hiking is not the only explanation for its presence in the French population. It further implies that migration at larger geographical scales, such as that between the UK and continental Europe [33,35], can influence local pathogen population structure and evolution.

For the *vir9 *gene, the story is more complex. After the resistance breakdowns in 1989 and 1994 and subsequent withdrawal of the susceptible cultivars, *vir9 *did not decrease in frequency, and currently all known *PST *pathotypes are fixed for this redundant virulence gene [48]. In part, this can be explained by its hitch-hiking with a newly arisen virulence gene (*vir17*, [34,35]), just like in the case of the *vir4 *gene. Moreover, we detected a fitness cost only for one *vir9 *isolate, while the other three isolates were even more competitive than their avirulent counterparts not carrying the *vir9 *gene. Our results further indicate that these differences are due to variation in aggressiveness and sporulation rates. The three successful isolates sporulate at high rates, while the less competitive one is among the least aggressive isolates of the *vir9 *collection.

Why is the *vir9 *gene costly in some isolates, but not in others? The answer may lie either in the gene itself or in its genetic background. First, in our four virulent isolates, the same *vir9 *virulence phenotype may represent different mutations, and only one imposes a fitness cost. Typically, virulence genes arise from mutations disrupting some metabolic function that would otherwise elicit a resistance reaction. It is conceivable that different mutations can bring about such modifications, and possibly not all of them come at a fitness cost [43]. Alternatively, costs of virulence may have been balanced by compensatory mutations. Compensation may occur directly in the gene(s) responsable for the loss of function, but also indirectly in genes determining pathogen aggressiveness. Indeed, aggressiveness is known to have a multigenic basis [49], thus offering multiple targets for compensatory mutations. If fitness effects are sufficiently large and selection efficient, this may even bring about a net positive effect and overcompensation of the cost of virulence. The history of the *vir9 *gene is compatible with such a scenario. Resistance breakdown of the *Yr9 *gene in England occurred already two years before highly aggressive *vir9 *variants appeared in France. Breakdowns are usually accompanied by massive PST epidemics, with exponential population growth and up to 10 infection cycles per growing season. This may have provided sufficient time for compensatory mutations to arise in the *vir9 *background and to be picked up by natural selection. Indeed, for the period in question, a signature of this process can be seen by the increase of the frequency of highly aggressive isolates in the *vir9 *spore collection. So far, cost-compensation has been described for resistance to antibiotics[50] or insecticides [51]. If our interpretations are correct, this study is one of the first to suggest the compensation of costs of virulence in natural populations of a pathogen. Similar ideas about the progressive evolution of the fitness of new pathotypes of cereal rusts date back to the 1970s. Based on empirical and experimental data, it was suggested that new phenotypes were unable to spread because of their low aggressiveness [52,53] and that adaptation to quantitative host resistance occurred gradually through polygenic changes [54].

Interestingly, we did not detect variation in neutral genetic markers among the isolates in the collection, despite the large number of primer combinations screened. As these isolates share the same clonal background, variation in both virulence and aggressiveness must have arisen within a relatively short time span. Indeed, Lande and Barrowclough [55] showed that in very large populations, heritable variation in quantitative traits can established by spontaneous mutation without leaving a trace in neutral genetic markers. Clearly, this rapid evolutionary change sets limits to our "all-else-being-equal" approach in the competition experiments, aiming at the comparison of isogenic isolates that differ only in the virulence gene in question.

## Conclusion

First, our study demonstrates substantial fitness costs of single virulence genes on different host varieties under field conditions. In part, these costs explain changes in the genetic composition of natural *PST *populations, consistent with van der Plank's hypothesis of selection against redundant virulence genes [21]. Second, however, we also found that redundant virulence genes are not automatically selected against, either because they do not have a fitness cost or because costs are compensated. Regardless of the precise mechanism, one of our main conclusions is that the fate of a particular virulence mutation may not be independent of its genetic background. This is particularly relevant for clonal pathogens, such as *PST*, where the whole genome is a single linkage group exposed to natural selection. Selection then perceives the sum of the costs of the different virulence genes carried by a given genotype and that of all other genes determining growth and transmission. Indeed, additional virulences may have more deleterious effects in genetic backgrounds that already carry other virulence genes [40,42,56] as it was the case in our study. The very different outcomes for the three virulence genes studied here indicate strong variation in the evolutionary trajectories of virulence mutations; this also stresses the need for more refined theoretical models to predict these trajectories.

Third, our results have implications for plant breeding strategies. Current large-scale programs employ inter-specific crosses and molecular engineering to develop resistance genes imposing a high cost of virulence for pathogens that overcome this resistance. This handicap may prevent the spread of such pathogen genotypes and thus increase the durability of resistance [57]. Obviously, this approach critically hinges on the consistency and durability of costs of virulence. Our experiments indicate that costs can be quite consistent, at least for certain virulence genes. However, durability of costs of virulence can be expected to be inversely proportional to the strength of selection imposed on the pathogen to compensate these costs. We suspect that that compensation may occur rather rapidly, by hitch-hiking processes or by efficient selection on multigenic traits. Thus, if demonstrating the cost of virulence is already complicated, predicting its durability may be even more difficult.

Finally, it is often argued that agro-patho-systems are too artificial or too simple to be comparable to natural host-parasite systems. Here, we demonstrated that the seemingly straightforward genetic architecture of the cultivated host produces complex patterns of genetic change in its natural opponent. These evolutionary changes can be investigated experimentally in real-time (several pathogen generations) and they remain traceable over larger time scales in the production areas (years or decades). We therefore believe that the study of these systems can provide valuable general insights in the costs and consequences of adaptation in host-parasite systems.

## Methods

### Species description

*Puccinia striiformis *f.sp. *tritici *(order *Uredinales*, class *Basidiomycetes*) is strongly wheat-specific (*Triticum aestivum *and *T. turgidum*). There is no known alternate host on which the sexual cycle could be completed and thus fungal reproduction is essentially asexual. Fungal mycelia systemically grow within leaf nervures (stripes); the asexual part of the life cycle is completed within only 14 days, resulting in repeated bursts of sporulating lesions releasing urediospores that infect neighbouring plants. Typically, these clonal fungal populations experience strong between-season bottlenecks every season, and from low initial genetic diversity, new virulence mutations arise during seasonal epidemics. In Europe, resistance breakdown usually takes only 3–4 years [35]. Even if treated the yield loss can reach 80% [58]. To date, a complete phylogeny of this species is still lacking, but introductions of new strains occur worldwide and can be attributed to both long distance wind dispersal and accidental human contamination.

### Competition experiments

We used 16 isolates collected as uredospores from infected fields in the North of France, between 1989 and 1997 (Table 1), and stored in liquid nitrogen. Virulence gene profiles were characterised on European and World differential cultivars, using Johnson *et al*.'s method [59]. We used 39 AFLP primer combinations to characterise phylogenetic relationships among the isolates [see Additional file 1]. Thus, competing pairs of *virulent *and *avirulent *isolates of the *vir9 *and *vir6 *pathotypes represented indistinguishable AFLP genotypes; for the *vir4 *pathotype, *competing *isolates differed by four AFLP bands and may therefore carry other mutations in addition to the virulence for *Yr4*.

We tested 8 virulent isolates, four carrying the *vir9 *virulence gene and two with the *vir4 *and *vir6 *virulence genes, respectively. Each *virulent *isolate was paired with an *avirulent *isolate as a competitor (Pairs *A-J*, Table 1). Most pairs were competed on two host cultivars and in two growing seasons (2005, 2006) under field conditions. Pairs *A *and *B *were also tested under climate chamber conditions. We established two independent replicates per pair, year and host cultivar.

### Preparation of inocula

To produce fresh inocula for the experiments, uredospores were taken out of the liquid nitrogen, heat shocked (40°C, 15 min) and used to inoculate 7-day-old seedlings of the susceptible cultivar *Victo*. Inoculated plants were incubated in a dew chamber at 8°C for 16 h in the dark to facilitate infection ([60] and then transferred to the greenhouse (day: 16 h, 350 μE·m^-2^·s^-1^, 17°C; night: 8 h, 14°C). Eighteen days after inoculation, spores were collected with a vacuum collector, dried in a desiccator for 3 days at 4°C and stored in microtubes at -80°C prior to the experiments.

### Climate chamber experiment (Pairs *A *and *B*, *vir9 *virulence gene)

For each replicate, a 50:50 mixture of 20 mg uredospores of the *virulent *and *avirulent *isolate was inoculated on approx. 400 7-day-old seedlings of a given host cultivar. We used two susceptible wheat varieties, *Thésée *(carrying the *Yr2 *resistance gene) and *Récital *(*Yr6*) [61]. We chose these cultivars because of their high susceptibility at the time of the appearance of the *vir9 *virulence in 1989. After incubation in a dew chamber at 8°C for 16 h, plants were placed in a climate chamber (day: 16 h, 350 μE·m^-2^·s^-1^, 17°C; night: 8 h, 14°C). Twenty days after inoculation, spores were collected and used to start a new infection cycle on the same host cultivar, as described above. We ran five cycles of competition and preserved a small sample of spores at each cycle to estimate the relative frequencies of the competing isolates.

### Field experiments

Field experiments were conducted during two wheat seasons in 2005 and 2006, at the Grignon Institute in Northern France (48°50'N 1°55'E). A replicate consisted of a 10 × 1.5 m plot, (250 plants/m^2^) of a given host cultivar. Experiments were started in late March, at the end of tillering. In the center of the plot, we planted two sporulating *Victo *seedlings that had been inoculated with a 50:50 mix of the two competing isolates, as described above. Preliminary tests showed that sporulation on the *Victo *seedlings did not significantly change the 50:50 ratio (data not shown). At the end of the epidemic (early June), uredospores were harvested and stored at -80°C until further processing. The absence of infection in surrounding plots indicated that natural or cross-contamination did not occur.

### Frequency assessment

Frequency assessments of the competing isolates followed the protocol by Lannou *et al. *(2005). For a given sample, we used an oil solution of 0.1 mg of the collected uredospores to inoculate five pots, each with 15 seedlings of a discriminating host cultivar. The cultivars *Sleipner *(*Yr9 *resistance gene), *Heines Kolben *(*Yr2, Yr6*) and *Hybrid 46 *(*Yr4*) were used for experiments with *vir9*, *vir6 *and *vir4 *virulence genes, respectively. On these discriminant cultivars, the virulent isolate develops sporulating lesions, whereas the avirulent isolate produces non-sporulating chloroses, indicating the failure of infection. Thus, the total number of chloroses was counted 7 days and sporulating lesions 14 days after inoculation. The frequency of the virulent isolate in the sample was taken as the ratio of sporulating chloroses/total chloroses. Each frequency was estimated on 400 chloroses on average. The goodness of the frequency assessment was previously validated for *Heines Kolben *and *Hybrid 46 *[62]. For *Sleipner*, we controlled the reliability of frequency estimates by the inoculation of different ratios of *Avirulent*/*virulent *isolates [100/0; 75/25; 50/50; 25/75 and 0/100, see Additional file 2].

### Estimation of the cost of virulence

We calculated the selective value (*s*) of the avirulent isolate from a model by Leonard [28], with Ln (q_n_/(1-q_n_)) = Ln (q_0_/(1-q_0_)) – n Ln (1-*s*); q_0 _is the initial frequency of the virulent isolate in the mix and q_n _its final frequency after *n *generations and (1-*s*) is the fitness difference between the virulent and the avirulent isolate. Following McGregor and Manners [63], the number of fungal generations *n *in the field experiments was estimated by fitting the duration of latent period (p) on the mean yearly temperature (T) at the Grignon site as: p = 28,24exp(-0,06T). From this, we obtained an estimate of five fungal generations in both years.

### Analysis of the collection of *vir9 *isolates

Of the 33 *vir9 *isolates analysed here, 11 were collected early after the first appearance of this pathotype in France (1989–1991). In 1992, this first epidemic was ended by a severe drought, and the remaining isolates were collected during the second epidemic period (1992–1997). The isolates all have the same virulence gene profile, as confirmed by previous analysis using the World and European differential cultivars as well as by our own tests using supplementary cultivars [*Federationx4Kavkaz *(*Yr9*), *Sleipner *(*Yr9+), Austerlitz *(*Yr6+*), *Early Premium*, *Anza*]. Isolates had been stored at -80°C and fresh uredospores were prepared as described above.

We used AFLP markers to investigate the neutral genetic diversity among the isolates. Ten combinations of *Pst*I and *Mse*I primers with two selective nucleotides were used. A subset of eight isolates was screened with 24 additional primer combinations with two selective nucleotides. The four isolates used in the competition experiments were tested with all 39 AFLP combinations. Genomic DNA extraction, DNA quantification on agarose gel and AFLP genotyping are described in detail by Enjalbert et *al*. [34,64].

Variation in aggressiveness was measured as follows. Seedlings of the *Clement *cultivar were grown in groups of five plants in 1 dm^3 ^pots in the greenhouse (14°C-8 h dark period and 17°C-16 h light period). When primary leaves were fully expanded, pots were exposed to a 16 h light regime and two pots per isolate were inoculated with 0.2 mg of uredospores, suspended in 300 μl of Soltrol [60,65]. After incubation in a dark dew chamber for 24 h at 8°C, seedlings were placed back in the greenhouse. Fifteen days after inoculation, the primary leaf of each seedling was examined visually for the infection type according to a scale from 0 (no symptoms) to 9 (high sporulation) [66].

To calibrate the infection type index, we measured the sporulation rate of 11 isolates, including those from the competition experiment (*i5*, *i6*, *i7*, *i8*). Groups of 10 *Clement *seedlings were grown in 5 cm^2 ^pots, as described above. At the 2-leaf stage (8 days), seedlings were thinned to 6 individuals per pot and exposed to a 16 h light regime. The second leaf from each seedling was removed and the first leaf attached horizontally, adaxial side up, on a Plexiglas sheet with plasticine (Ulmann, Paris, France) [60]. Seedlings from 10 pots were inoculated together in a settling tower [67], with 5 mg spores of a given isolate (≈ 52 spores deposited per cm^2^). After incubation, seedlings were kept in a climate chamber, as described above. Eight days after their opening, we counted the number of lesions and estimated the inoculated part of the leaf surface estimated (width × length) on two leaves per pot, from which we calculated the lesion density per cm^2^. The pots were then placed individually under plastic jars to prevent cross-contamination. Uredospores were collected 17 and 22 days after inoculation, dried for 24 h at 5°C and then weighed. For each pot, we calculated sporulation rate as the ratio of spore production (mg) over lesion density.

### Statistical analyses

Changes in (arcsine-transformed) frequency of virulent isolates in the climate chamber competition experiment were analysed by means of a repeated measure ANOVA, using the MIXED procedure of SAS statistical software [68]. Variation in final frequencies of virulent isolates in the field competition experiments were analysed by means of Analysis of Deviance, based on a logistic regression (GENMOD procedure; SAS). Fully factorial statistical models contained the identity of competing pairs (Pair *A*, *B*, etc.), host cultivar and year. In all analyses, frequencies for a given replicate represented combined values over all pots and plants of the discriminating cultivar. We further used *t*-tests to test whether the final frequency of virulent isolates was significantly different from 0.5 across all replicates and pairs. Frequencies in the 1:1 mixtures at the beginning of the experiments were not significantly different from 0.5 (not shown). One-way ANOVAs were employed to compare differences in mean and variance of infection type among isolates from the two epidemic periods.

## Authors' contributions

BB carried out the study in controlled conditions and field experiments. BB, OK and JE analyzed the results, performed the statistical analysis and drafted the manuscript. ML participated in the design and coordination of the experiments. JE and CP designed the study and CP helped to draft the manuscript. All authors read and approved the final manuscript.

## Supplementary Material

Additional file 1 **Phylogenetic relationships between *PST *isolates used in competition.** The table (a) shows the list of the 39 primer combinations used for AFLP analysis. The most parsimonious tree (b), built on the basis of AFLP polymorphism, revealed low divergence between two clonal lineages, while within each lineage, no molecular divergence was found between isolates differing by a single virulence. Each race is coded by its combination of virulences against corresponding specific resistance genes (*Yr1*, *Yr2 *and so on). Click here for file

Additional file 2 **Validation of the use of *Sleipner *cultivar to assess *vir9 *proportion in spore mixtures.** The figure shows the reliability of frequency estimates on cv. *Sleipner *after inoculation with different ratios of *Avir9*/*vir9 *isolates. For two independent pairs of *Avir9/vir9 *isolates, we prepared spore mixtures containing different proportions of the *vir9 *isolate: 0%, 25%, 50%, 75% and 100%. For each isolate pair and proportion, two independent inoculations were performed over 5 pots, each containing 10 to 15 seedlings. After 10 days, the frequency of the virulent isolate was measured (see Methods, "frequency assessment"). Click here for file
