# Supplementary material for: Tracking costs of virulence in natural populations of the wheat pathogen, Puccinia striiformis f.sp.tritici
Source: BMC Evol Biol. 2009 Jan 30;9:26. doi: 10.1186/1471-2148-9-26 (PMC2660305; doi:10.1186/1471-2148-9-26)
Supplement: Additional file 1 — Phylogenetic relationships between PST isolates used in competition. The table (a) shows the list of the 39 primer combinations used for AFLP analysis. The most parsimonious tree (b), built on the basis of AFLP polymorphism, revealed low divergence between two clonal lineages, while within each lineage, no molecular divergence was found between isolates differing by a single virulence. Each race is coded by its combination of virulences against corresponding specific resistance genes (Yr1, Yr2 and so on). [file 1471-2148-9-26-S1.doc]

Title: Phylogenetic relationships between *PST* isolates used in competition

Description: The table (a) shows the list of the 39 primer combinations used for AFLP analysis. The most parsimonious tree (b), built on the basis of AFLP polymorphism, revealed low divergence between two clonal lineages, while within each lineage, no molecular divergence was found between isolates differing by a single virulence. Each race is coded by its combination of virulences against corresponding specific resistance genes (*Yr1*, *Yr2* and so on).

(a)

# £ P(AA) and M(AA) are corresponding reciprocally to a *Pst* primer (5’GTAGACTGCGTACATGCAG) and a *Mse* primer (5’GACGATGAGTCCTGAGTAA) plus two selective nucleotides, respectively AA and AA, and followed by their code

(b)

**Additional data file 2. Validation of the use of *Sleipner* cultivar to assess *vir9* proportion in spore mixtures**. For two independent pairs of *Avir9/vir9* isolates, we prepared spore mixtures containing different proportions of the *vir9* isolate: 0%, 25%, 50%, 75% and 100%. For each isolate pair and proportion, two independent inoculations were performed over 5 pots, each containing 10 to 15 seedlings. After 10 days, the frequency of the virulent isolate was measured as described in the Material and Methods (section "frequency assessment").
