# Supplementary material for: Tracking costs of virulence in natural populations of the wheat pathogen, Puccinia striiformis f.sp.tritici
Source: BMC Evol Biol. 2009 Jan 30;9:26. doi: 10.1186/1471-2148-9-26 (PMC2660305; doi:10.1186/1471-2148-9-26)
Supplement: Additional file 2 — Validation of the use of Sleipner cultivar to assess vir9 proportion in spore mixtures. The figure shows the reliability of frequency estimates on cv. Sleipner after inoculation with different ratios of Avir9/vir9 isolates. For two independent pairs of Avir9/vir9 isolates, we prepared spore mixtures containing different proportions of the vir9 isolate: 0%, 25%, 50%, 75% and 100%. For each isolate pair and proportion, two independent inoculations were performed over 5 pots, each containing 10 to 15 seedlings. After 10 days, the frequency of the virulent isolate was measured (see Methods, "frequency assessment"). [file 1471-2148-9-26-S2.doc]

Title: Validation of the use of *Sleipner* cultivar to assess *vir9* proportion in spore mixtures

Description: The figure shows the reliability of frequency estimates on cv. *Sleipner* after inoculation with different ratios of *Avir9*/*vir9* isolates. For two independent pairs of *Avir9/vir9* isolates, we prepared spore mixtures containing different proportions of the *vir9* isolate: 0%, 25%, 50%, 75% and 100%. For each isolate pair and proportion, two independent inoculations were performed over 5 pots, each containing 10 to 15 seedlings. After 10 days, the frequency of the virulent isolate was measured (see Methods, "frequency assessment").
